# Resistance Tracking Control of Memristors Based on Iterative Learning

**DOI:** 10.3390/e25050774

**Published:** 2023-05-10

**Authors:** Wei Cao, Jinjie Qiao

**Affiliations:** 1 College of Computer and Control Engineering, Qiqihar University, Qiqihar 161006, China; 2 College of Economics and Management, Qiqihar University, Qiqihar 161006, China; 01469@qqhru.edu.cn

**Keywords:** memristor, iterative learning control, cubic nonlinear, resistance tracking

## Abstract

A memristor is a kind of nonlinear two-port circuit element with memory characteristics, whose resistance value is subject to being controlled by the voltage or current on both its ends, and thus it has broad application prospects. At present, most of the memristor application research is based on the change of resistance and memory characteristics, which involves how to make the memristor change according to the desired trajectory. Aiming at this problem, a resistance tracking control method of memristors is proposed based on iterative learning controls. This method is based on the general mathematical model of the voltage-controlled memristor, and uses the derivative of the error between the actual resistance and the desired resistance to continuously modify the control voltage, making the current control voltage gradually approach the desired control voltage. Furthermore, the convergence of the proposed algorithm is proved theoretically, and the convergence conditions of the algorithm are given. Theoretical analysis and simulation results show that the proposed algorithm can make the resistance of the memristor completely track the desired resistance in a finite time interval with the increase of iterations. This method can realize the design of the controller when the mathematical model of the memristor is unknown, and the structure of the controller is simple. The proposed method can lay a theoretical foundation for the application research on memristors in the future.

## 1. Introduction

In 1971, American scientist Chua first proposed the concept of a memristor based on the completeness of circuit theory [1]. Since at that time the existence of the memristor was only speculated mathematically, and it was not physically implemented yet, it has not attracted considerable attention during the more than three decades after it was proposed. Until May 2008, the nanometer memristor was successfully developed by the United States’ Hewlett-Packard (HP) laboratory to develop a physical model, and the results of this research were published in “Nature” [2,3]. Since then, there has been a wave of research on memristors around the world [4]. Essentially, a memristor is a nonlinear resistor with a memory function. Its resistance value is controlled by the voltage or current added to both ends, it can maintain the previous value in the case of a power failure, and it will be cleared only when the current is in the opposite direction. Therefore, it has broad application prospects in many fields.

Up to now, scholars at home and abroad have mainly studied memristors from three aspects. The first is how to develop a device with memristive characteristics that is more economical and easy to implement, using advanced materials based on the idea of the memristive physical model by HP Labs. According to different materials, it can be divided into several typical memristors, such as thin film memristors [2], spin memristors [5], three-terminal memristors [6], phase transition memristors [7], and so on. The second aspect is to analyze the dynamic behavior of a memristor and the memristive system by following the thinking of Professor Chua. For example, the supertrack function of Chua’s oscillator and Chua’s circuit with a memristor is calculated by using the supertrack method, and the behavior of the system is explained by such a supertrack function in [8]. A memristor model with negative resistance is proposed in [9]. This model breaks the resistance state polarity limitation of the original memristor and provides a richer variation performance for the memristor to act as a neural network synaptic bionic device. The negative-type locally active memristor (N-type LAM) neuron circuit is designed by revealing the dynamic characteristics of LAM in [10]. The dynamic behavior of the circuit is studied quantitatively by means of Hopf bifurcation and numerical analysis, and a variety of neurmorphic behaviors are simulated successfully. The third aspect refers to the relative application studies of memristors. This aspect is studied mainly based on the various models of memristors and the nonlinear, nonvolatile, or switching characteristics of memristors [11,12,13,14,15,16,17,18,19,20,21,22,23].

The application research on memristors is mainly reflected in the fields of nonvolatile memory [11], chaotic circuits [12,13], artificial neural networks [14,15], and analog circuits [16,17]. The weights in the artificial neural network are realized by the multiplier, and this will lead to two defects. One is that the multiplier must be replaced again when the neural network needs to be modified, the other is that it is relatively large. If the weight of the neural network is realized by using the memristor as the electronic synapse, it is easy to achieve the purpose of modifying the neural network only by applying the external voltage source to change the resistance of the memristor. Based on this, a recurrent neural network model is established based on memristors, and the sufficient conditions of global uniformly asymptotical stability of recurrent neural network are obtained [18]. A neural network proportional-integration-derivative (PID) controller based on the memristor is designed by using the nonlinear characteristics and memory function of the memristor [19]. The weight update of the neural network is realized by the memristor, which simplifies the update algorithm of the network weight and lays the foundation for the realization of the new intelligent PID controller. The memristive PID controller is designed by replacing the resistance in the traditional PID controller hardware circuit with a memristor and using an additional control circuit to realize the adjustment of the memristor [20]. The designed controller achieves the goal of self-modification of the PID control parameters and thus can improve the control precision. A gain-adjustable amplifier circuit based on memristors is designed by changing the resistance of memristor [21,22]. The change of memristor is realized by changing the frequency and the pulse width of the control input signal. In general, when the basic arithmetic operations of the signals are implemented by the analog circuits, they are all represented by the voltage or current. The memristor can replace the voltage or current that represents the arithmetic operation [23]. The precision of this method is verified by the simulation experiments.

Throughout the literature on the application research on memristors, it can be found that the goals of the research are achieved by changing the resistance of the memristor. This involves how to change the resistance of the memristor and whether the resistance of the memristor can be changed according to the desired resistance. Therefore, for the two problems, the paper proposes a resistance tracking control method of memristors based on iterative learning control.

The idea of iterative learning control (ILC) is for a controlled system with repetitive operation characteristics, the current control input is continuously modified by using the deviation between the actual output and the desired output until the control input converges to the desired control, thereby achieving the goal of ensuring the actual output complete tracking of the desired output [24]. Iterative learning control has a strong ability to handle nonlinear dynamic systems, and can suppress all interference signals that repetitively appear. At the same time, the design of the controller does not depend on the precise model information of the controlled system, and its simple structure is convenient for engineering implementation. Therefore, iterative learning control has achieved fruitful research results after more than 30 years of development [25,26,27,28].

In view of the above analysis, in order to further broaden the application range of memristors and lay the foundation for future application research on memristors, this paper proposes an iterative learning control method for memristors by analyzing the general mathematical model of memristors. In this method, the resistance of the memristor is output, and the resistance change rate is the state equation. The derivative of the error between the actual resistance and the desired resistance is used to continuously modify the control voltage of the last memristor, so that making the current control voltage gradually approach the desired control voltage, thereby achieving the purpose of changing the memristor according to the desired resistance. Furthermore, the convergence condition of the algorithm is theoretically derived, and it is proved that the proposed method can make the memristor completely track the desired resistance within a finite time interval with the increase of iterations. Finally, the simulation examples are given to further validate the effectiveness of the proposed algorithm.

## 2. The Model of Memristors

**Figure 1:**
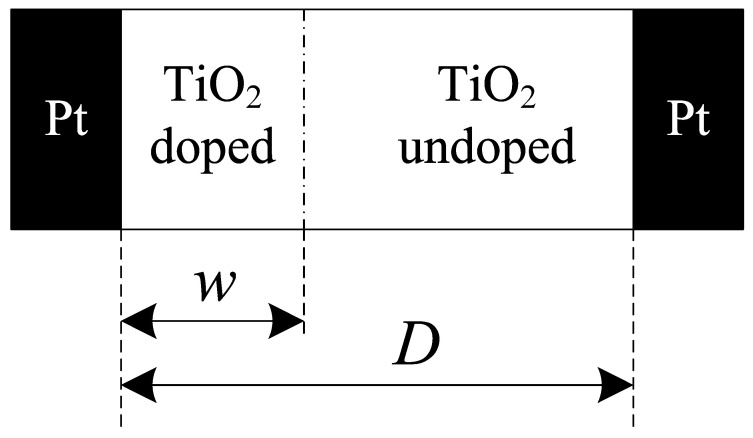
The HP memristor model. where w is the width of the conduction layer and D is the total width of the TiO_2_ film (about 10 nm).

In 2008, the research team of Hewlett-Packard Lab successfully created a physical model of the memristor using thin TiO_2_ film and Pt metal. The model is composed of a thin titanium dioxide (TiO_2_) film and two pieces of platinum (Pt). The titanium dioxide film sandwiched between two pieces of platinum is divided into two parts: one is the insulating layer without impurities (nondoping region), the other is the conduction layer after doping impurities (doping region). Its structure is shown in Figure 1.

When a voltage is applied to both ends of the memristor, the oxygen vacancy in the titanium dioxide film will drift under the action of the electric field. When the applied voltage makes the oxygen vacancy move from the conduction layer to the insulating layer, the conduction layer becomes wider and the insulating layer narrows gradually, so that the resistance value of the memristor will decrease; when the applied voltage makes the oxygen vacancy move from the insulating layer to the guide layer, the insulating layer becomes wider and the conduction layer narrows gradually, so that the resistance value of the memristor will increase. According to this principle and Figure 1, the voltage at both ends of the memristor can be obtained as follows.

(1) v(t)=M(z)i(t)

(2) $$M(z)=R_{\mathrm{on}}z+R_{\mathrm{off}}(1-z)$$

where i(t) is the current flowing through the memristor, z=w/D , z∈(0,1) is the boundary position of the insulation layer and the conduction layer, M(z) is the resistance value of the memristor, $R_{\mathrm{on}}$ is the resistance value of the memristor when the titanium dioxide film is all the conduction layer, and $R_{\mathrm{off}}$ is the resistance value of the memristor when the titanium dioxide film is all insulating layer.

Under the action of the electric field, the relationship between the ion drift rate and the change rate of conduction layer width is as follows:

(3) $$\frac{dw}{dt}=\mu_{v}\frac{R_{\mathrm{on}}}{D}i(t)$$

where $\mu_{v}=10^{-14}\;m^{2}\cdot s^{-1}\cdot v^{-1}$ is the drift rate of the ions in the uniform field. Therefore, the drift velocity of the interface can be obtained according to (3) and z=w/D:

(4) $$\frac{dz}{dt}=\mu_{v}\frac{R_{\mathrm{on}}}{D^{2}}i(t)=ki(t)$$

Since for the fixed model $k=\mu_{v}\frac{R_{\mathrm{on}}}{D^{2}}$ is a constant, it can be seen from (4) that the ion drift is linear. However, the memristor is a nanometer device, and a small voltage can produce a strong electric field, so as to lead to the nonlinear drift of ions, which is more significant at the edge of the device. In order to characterize the nonlinear drift of ions, it is necessary to multiply a window function g(z) [29] on the right side of (4), and then we obtain:

(5) $$\frac{dz}{dt}=ki(t)g(z)$$

Equation (4) is also called a linear impurity drift model, and (5) is the window function model. In addition, a piecewise function model [30] and a cubic nonlinear model [31] are often used. The advantages and disadvantages of these four commonly used models can be directly referred to [32].

In [33], the researchers presented general mathematical models of a voltage-controlled memristor.

(6) $$\dot{M}(t)=f(M,v,t)$$

(7) $$i(t)=M^{-1}v(t)$$

where M is the resistance value of the memristor, f is the nonlinear function, and $M^{-1}=1/M$ is the memory conductance of the memristor.

Since the memristor is a nano-sized circuit component, it has not been marketed due to the limitations of nano-technology. Therefore, the research into memristors is mainly to establish the mathematical model of memristors [34,35], the equivalent circuit model [36,37], and the simulation model [38,39]. The application research for memristors is mainly based on various models of memristors and the nonlinear, nonvolatile, or switching characteristics of memristors.

## 3. The Control Method Design of Memristors

In order to further broaden the application range of memristors, this paper designs an iterative learning control method by analyzing the mathematical model of voltage-controlled memristors. This method makes the input voltage of the memristor approach the desired input voltage gradually with the increase of iterations, so that the resistance value of the memristor gradually approaches the desired resistance value.

In this paper, let the output of the memristor be y(t)=x(t), where x(t) is the resistance value of the memristor, let the input voltage of the memristor be v(t) as the control variable, f(x,v,t) is the nonlinear function of v(t) and x(t), and assuming that the k-th iteration is currently performed and the time interval of repetitive operation is t∈[0,T], then the voltage-controlled memristor can be expressed as follows:

(8) $$\left\{\begin{array}{l} {\dot{x}}_{k}(t)=f(x_{k}(t),v_{k}(t),t)\\ y_{k}(t)=x_{k}(t) \end{array}\right.$$

where the subscript k indicates the number of the iterative learnings.

**Assumption 1.**  *There exists the partial derivative *$A_{k}$*,* $B_{k}$ *of the nonlinear function *$f(x_{k}(t),v_{k}(t),t)$ *with respect to* x*,* v*, and* $A_{k}$*,* $B_{k}$ *are differentiable with respect to *t *and *$B_{k}$ *is bounded, where *$A_{k}=\frac{\partial f}{\partial x}\left|\begin{array}{l} {}_{v=\eta_{k}^{\prime }}^{x=\xi_{k}^{\prime }} \end{array}\right.$*, *$B_{k}=\frac{\partial f}{\partial v}\left|\begin{array}{l} {}_{v=\eta_{k}^{\prime }}^{x=\xi_{k}^{\prime }} \end{array}\right.$*, *$A_{k}$ *and *$B_{k}$ *are functions of* $\xi_{k}^{\prime }$*,* $\eta_{k}^{\prime }$*,* t*. According to the differential median theorem,* $\xi_{k}^{\prime }$ *is a resistance between* $x_{k}(t)$ *and* $x_{k+1}(t)$*, and* $\eta_{k}^{\prime }$ *is a voltage between* $v_{k}(t)$ *and* $v_{k+1}(t)$.

**Assumption 2.**  *The nonlinear function *$f(x_{k}(t),v_{k}(t),t)$ *is reversible. That is, for a given desired resistance *$x_{d}(t)$*, there is a unique desired control voltage *$v_{d}(t)$*, which can make the state of the system be the desired value, i.e., satisfying*

(9) $${\dot{x}}_{d}(t)=f(x_{d}(t),v_{d}(t),t)$$

**Assumption 3.**  *At each operation, the system satisfies *$x_{d}(0)=x_{k}(0)$.

**Remark 1.**  *Assumption 2 is the controllability condition of a given trajectory. If the condition is not satisfied, the control of the system will be meaningless. Assumption 3 requires that each iteration satisfies the same initial condition, which is the basic condition to realize the perfect tracking control. In practice, this assumption may not be strictly satisfied, but it can be made consistent with the actual value by adjusting the initial value of the desired trajectory.*

**Figure 2:**
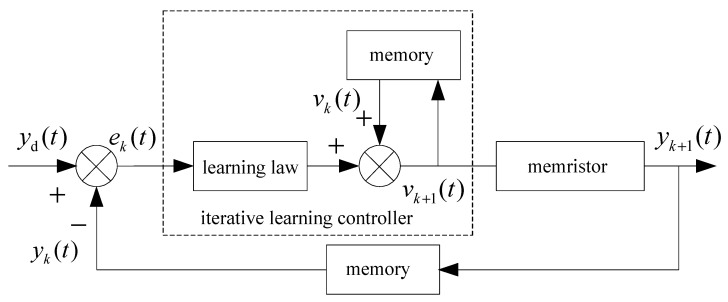
System structure of the iterative learning control.

The paper uses the derivative-type (D-type) iterative learning control method to control the resistance of the memristor. Then the control algorithm is designed as follows:

(10) $$v_{k+1}(t)=v_{k}(t)+L{\dot{e}}_{k}(t)$$

where L is a learning gain, $e_{k}(t)=y_{d}(t)-y_{k}(t)$ is a tracking error of the resistance. The system structure figure of the iterative learning control is shown as Figure 2.

The control objective of this paper is to use the algorithm (10) to control the nonlinear system (8) satisfying the Assumptions 1–3, so that the actual output $y_{k}(t)$ of the system can gradually track the desired output $y_{d}(t)$ with the increase of iterations in a finite time interval.

## 4. Convergence Analysis of the Control Algorithm

For the convenience of the subsequent convergence proof, the following related definitions are first given here.

**Definition 1**  [40]**.** *The* λ *norm of a vector function* h:[0,T] $\to R^{n}$ *is defined as*

$${\left\|h\right\|}_{\lambda }=\underset{t\in [0,T]}{\sup}\left\{e^{-\lambda t}\left\|h(t)\right\|\right\},\;(\lambda >0)$$

where $\left\|\cdot \right\|$ is a norm on $R^{n}$.

The main results of this paper are given below.

**Theorem 1.**  *The iterative learning control algorithm (10) is adopted to control the nonlinear system (8) satisfying the Assumptions 1–3. When selecting the bounded learning gain L* *, satisfying*

(11) $$\left\|I-B_{k}L\right\|=\rho <1$$

then as k→∞, the output $y_{k}(t)$ converges to the desired trajectory $y_{d}(t)$, i.e., $\underset{k\to \infty }{\lim}y_{k}(t)=y_{d}(t)$, which means the memristive resistance $x_{k}(t)$ can converge to the desired resistance $x_{d}(t)$. Among them, the definition of $B_{k}$ can be found in Assumption 1 above.

**Proof of Theorem 1.**  *According to the definition of the tracking error and Assumption 3, we find*

(12) $$\begin{array}{cc} e_{k+1}(t) & =y_{d}(t)-y_{k+1}(t)\\ & =y_{d}(t)-y_{k}(t)+y_{k}(t)-y_{k+1}(t)\\ & =e_{k}(t)-(x_{k+1}(t)-x_{k}(t))\\ & \;\;=e_{k}(t)-\int_{0}^{t}f(x_{k+1}(\tau ),u_{k+1}(\tau ),\tau )d\tau +\int_{0}^{t}f(x_{k}(\tau ),u_{k}(\tau ),\tau )d\tau . \end{array}$$

Since it is difficult to obtain the analytic expression of the system (8), the convergence condition of the iterative learning control algorithm (10) cannot be obtained directly from (12). As a result, the following processing is needed.

Let $\delta x_{k}=x_{k+1}-x_{k}$, $\delta u_{k}=u_{k+1}-u_{k}$, $x_{k+1}$ expand at $x_{k}$, then by (8), we obtain

(13) $$\delta {\dot{x}}_{k}=A_{k}\delta x_{k}+B_{k}\delta u_{k}$$

Combining with (13), (12), (10), and Assumption 3 yields:

(14) $$\begin{array}{cc} e_{k+1}(t) & =e_{k}(t)-\int_{0}^{t}\Phi_{k}(t,\tau )B_{k}\left[u_{k+1}(\tau )-u_{k}(\tau )\right]d\tau \\ & =e_{k}(t)-\int_{0}^{t}\Phi_{k}(t,\tau )B_{k}L{\dot{e}}_{k}(\tau )d\tau \\ & =e_{k}(t)-\Phi_{k}(t,t)B_{k}Le_{k}(t)\\ & +\Phi_{k}(t,0)B_{k}Le_{k}(0)+\int_{0}^{t}\frac{\partial [\Phi_{k}(t,\tau )B_{k}L]}{\partial \tau }e_{k}(\tau )d\tau \\ & =e_{k}(t)-B_{k}Le_{k}(t)+\int_{0}^{t}\frac{\partial [\Phi_{k}(t,\tau )B_{k}L]}{\partial \tau }e_{k}(\tau )d\tau \\ & \;\;\;=\left(I-B_{k}L\right)e_{k}(t)+\int_{0}^{t}\frac{\partial [\Phi_{k}(t,\tau )B_{k}L]}{\partial \tau }e_{k}(\tau )d\tau , \end{array}$$

where $\Phi_{k}(t,\tau )$ simply represents the state transition matrix $\Phi_{k}(\xi_{k}^{\prime },\eta_{k}^{\prime },t,\tau )$ of Equation (13). Taking norm of both sides of Equation (14), we derive:

(15) $$\left\|e_{k+1}(t)\right\|\leq \left\|I-B_{k}L\right\|\left\|e_{k}(t)\right\|+\int_{0}^{t}\left\|\frac{\partial [\Phi_{k}(t,\tau )B_{k}L]}{\partial \tau }\right\|\left\|e_{k}(\tau )\right\|d\tau$$

Multiplying both sides of Equation (15) by $e^{-\lambda t}$, where λ>0, then there exists

(16) $$e^{-\lambda t}\left\|e_{k+1}(t)\right\|\leq \left\|I-B_{k}L\right\|e^{-\lambda t}\left\|e_{k}(t)\right\|+b_{1}\int_{0}^{t}e^{-\lambda (t-\tau )}e^{-\lambda t}\left\|e_{k}(\tau )\right\|d\tau$$

where $b_{1}=\underset{t\in [0,T],\tau \in [0,t]}{\sup}\left\|\frac{\partial [\Phi_{k}(t,\tau )B_{k}L]}{\partial \tau }\right\|$. According to the definition of λ norm, by Equation (16) we have

(17) $${\left\|e_{k+1}\right\|}_{\lambda }\leq \rho {\left\|e_{k}\right\|}_{\lambda }+b_{1}\frac{1-e^{-\lambda T}}{\lambda }{\left\|e_{k}\right\|}_{\lambda }=\left(\rho +b_{1}\frac{1-e^{-\lambda T}}{\lambda }\right){\left\|e_{k}\right\|}_{\lambda }=\bar{\rho }{\left\|e_{k}\right\|}_{\lambda }$$

where $\bar{\rho }=\rho +b_{1}\frac{1-e^{-\lambda T}}{\lambda }$. From the convergence condition (11) in Theorem 1, we know 0<ρ<1. It is possible to choose a sufficiently large λ so that $0<\bar{\rho }<1$ holds. Therefore, we know from Equation (17) there exists $\underset{k\to \infty }{\lim}{\left\|e_{k}\right\|}_{\lambda }=0$, that is, $\underset{k\to \infty }{\lim}x_{k}(t)=x_{d}(t)$. As such, we have completed the proof of Theorem 1. □

## 5. The Simulation Experiments

**Figure 3:**
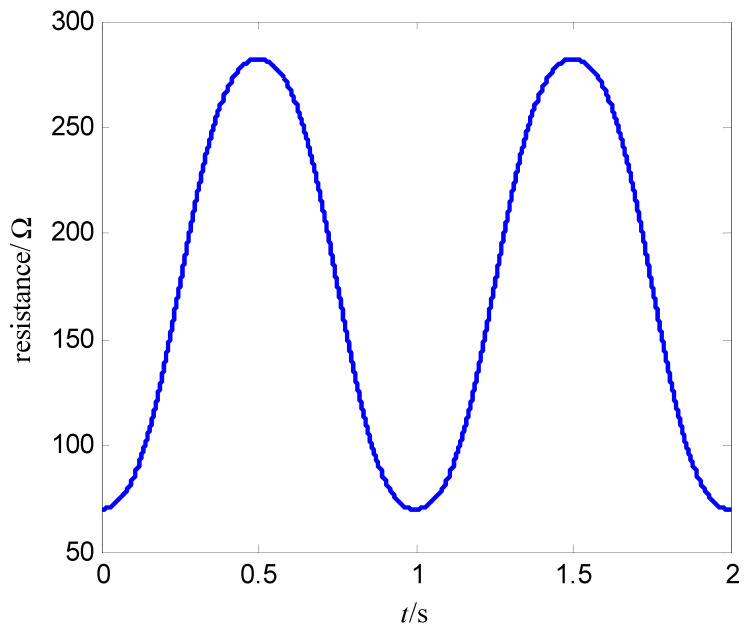
Resistance−change curve of the memristor.

**Figure 4:**
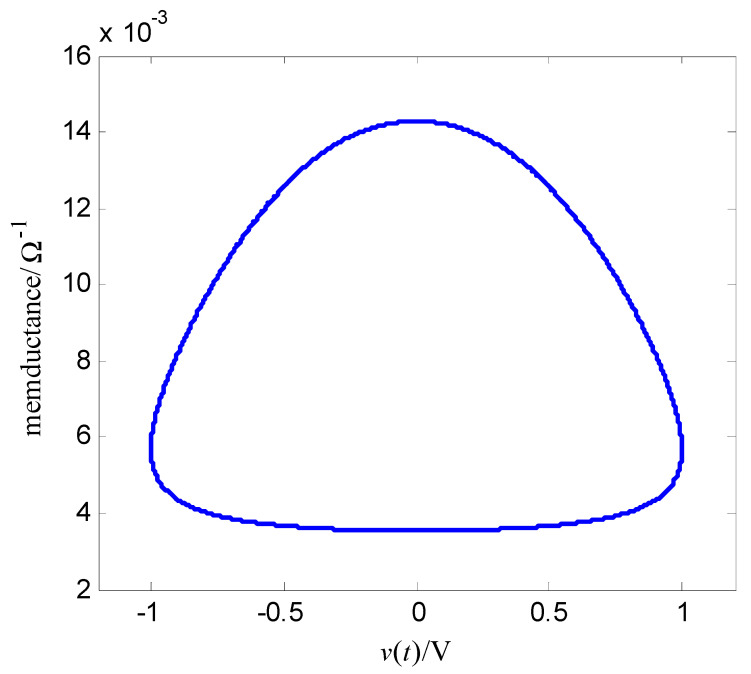
Voltage−memductance curve of the memristor.

**Figure 5:**
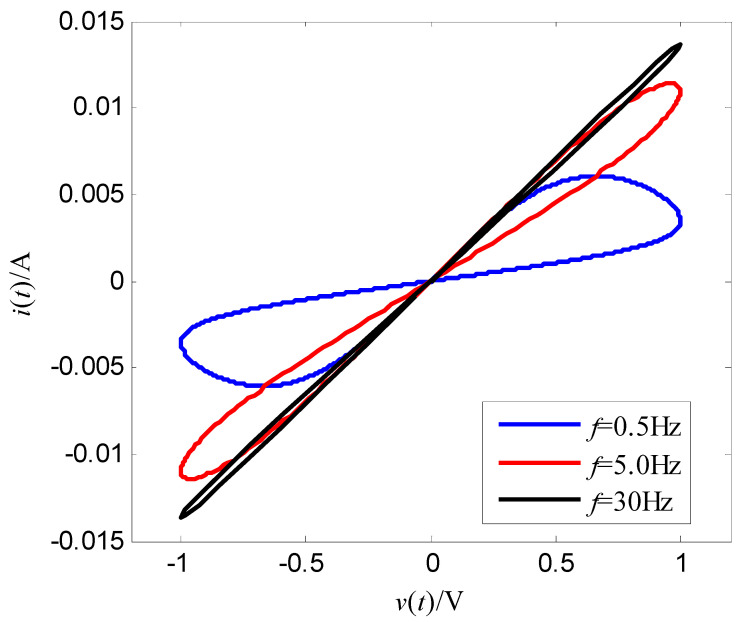
Effect of the voltage frequency on the hysteresis loop.

**Figure 6:**
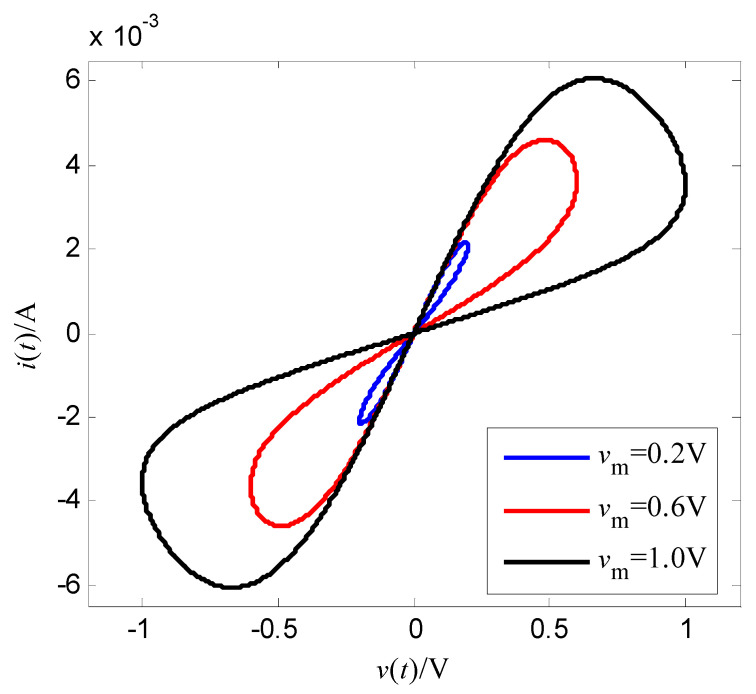
Effect of the voltage amplitude on the hysteresis loop.

In order to further verify the effectiveness of the proposed algorithm, the simulation experiments were performed on the following memristors. According to the cubic nonlinear model of the memristor proposed in reference [31], combining with the general mathematical model of the voltage-controlled memristor, the resistance change rate of the memristor in the paper is expressed as a cubic nonlinear function of the input voltage. Its mathematical expression is shown as follows:

(18) $$\dot{x}(t)=f(x,v,t)=\alpha v+\beta v^{3}$$

where α and β are constant, v is the input voltage (control voltage), x is the resistance of the memristor, and f(x,v,t) is the cubic nonlinear function of the input voltage. Let the memductance be $W(x,v,t)=x^{-1}$, then the output current of the memristor is $i(t)=x^{-1}v(t)$. This expression (18) is a relatively smooth mathematical model of a nonlinear function f(x,v,t). Select α=400 Ω/(V⋅s), $\beta =400\;\Omega /(V^{3}\cdot s)$, the initial value of memristor is x(0)=70 Ω, the magnitude of the sinusoidal AC voltage of the memristor is $v_{m}$, the frequency is f, i.e., $v(t)=v_{m}\sin(2\pi ft)$. As t=2 s, $v_{m}=1.0\;V$, f=1.0 Hz, Figure 3 is the change curve of the resistance of the memristor with time, and Figure 4 is voltage-memductance curve of memristor. Assuming $v_{m}=1.0\;V$ stays fixed, when f is respectively chosen as 0.5 Hz, 5 Hz, 30 Hz, the voltage-current characteristic curves of memristor are shown in Figure 5; assuming f=0.5 Hz stays fixed, when $v_{m}$ is respectively chosen as 0.2 V, 0.6 V, 1.0 V, the voltage-current characteristic curves of memristor are shown in Figure 6. It can be seen from Figure 2 to Figure 6 that the resistance of the memristor (18) has nonlinear characteristics, and the hysteresis loop of the memristor (18) can gradually narrow with the increase of frequency and widen with the increase of amplitude, that is, Equation (18) accords with the basic characteristics of the memristor.

In order to verify the control effect of the proposed algorithm (10) on the memristor resistance, Equation (18) is used to represent the resistance change rate of the memristor. Let the resistance $x_{k}(t)$ be the output $y_{k}(t)$, and $v_{k}(t)$ be the control voltage. Select α=400 Ω/(V⋅s), $\beta =400\;\Omega /(V^{3}\cdot s)$, the iterative learning gain is L=0.0001, the control voltage at first iteration is $v_{0}(t)=0$, the simulation time is T=4 s, the sampling period is 0.01 s, then the expected resistance of the memristor is divided into the following two cases:

(19) $$\begin{array}{l} y_{d1}(t)=x_{d1}(t)=260+240\sin(\pi t)\\ \;\;\;\;x_{1}(0)=x_{d1}(0)=260\;\Omega \end{array}$$

(20) $$\begin{array}{l} y_{d2}(t)=x_{d2}(t)=\left\{\begin{array}{l} 1000t,\;\;\;\;\;\;0\leq t<1\\ 1000,\;\;\;\;\;\;\;\;1\leq t\leq 2\\ -500t+2000,\;\;\;2<t<3\\ 500,\;\;\;\;\;\;\;\;\;\;3\leq t\leq 4 \end{array}\right.\\ \;\;\;\;\;\;\;x_{2}(0)=x_{d2}(0)=0\;\Omega \end{array}$$

**Figure 7:**
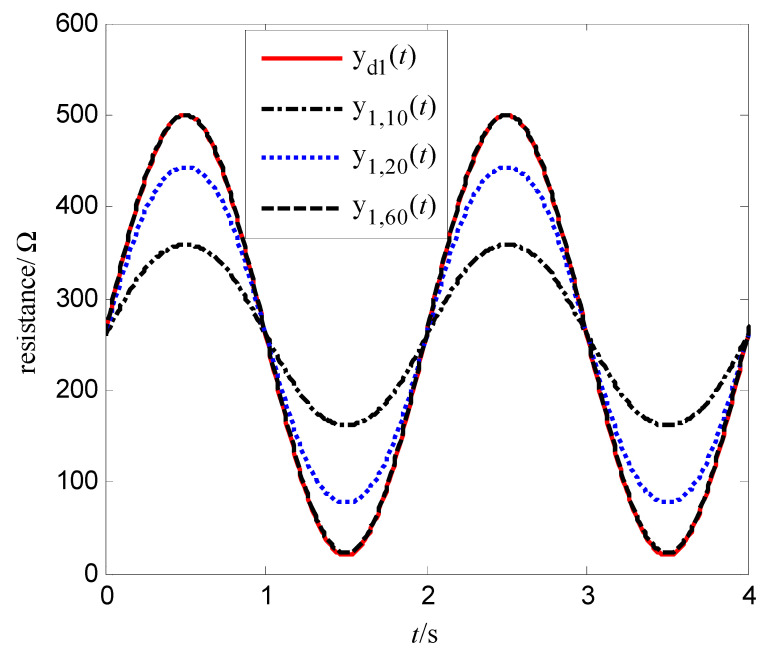
Tracking of the resistance to Equation (19).

**Figure 8:**
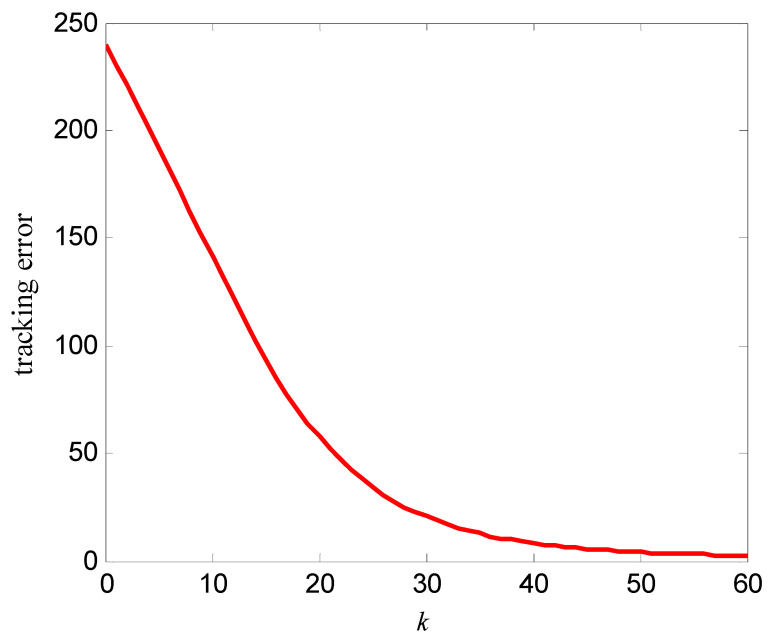
Tracking error of the resistance to Equation (19).

**Figure 9:**
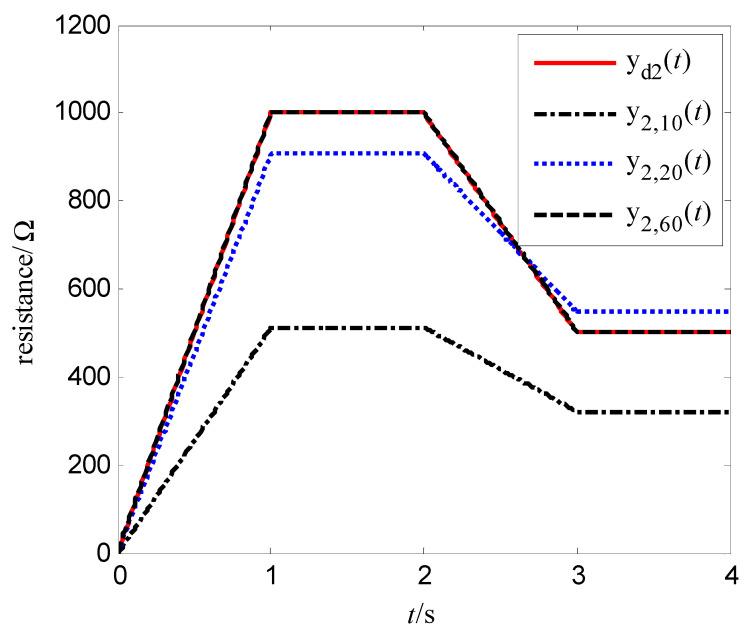
Tracking of the resistance to Equation (20).

**Figure 10:**
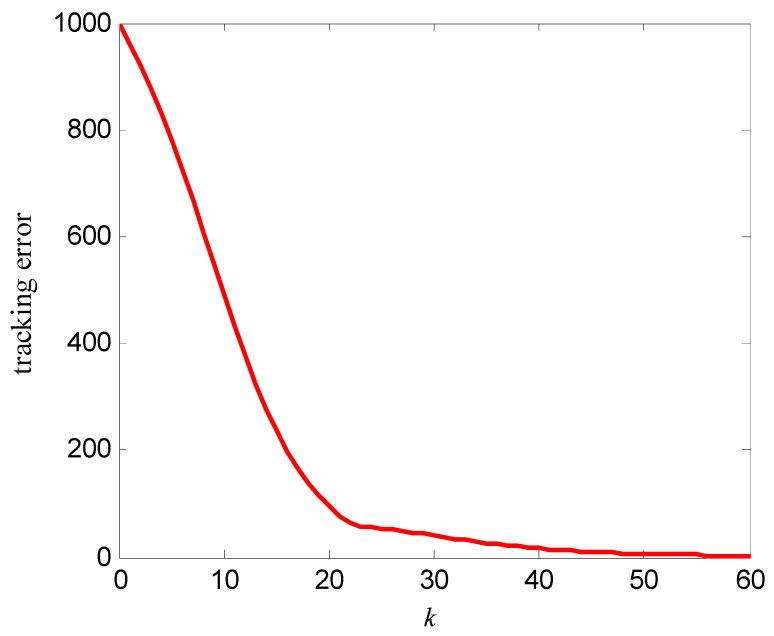
Tracking error of the resistance to Equation (20).

The simulation results are shown in Figure 7, Figure 8, Figure 9 and Figure 10. Figure 7 is the tracking curve of the memristor’s resistance to Equation (19) at different iterations, Figure 8 is the tracking error curve of the memristor’s resistance to Equation (19), Figure 9 is the tracking curve of the memristor’s resistance to Equation (20) at different iterations, and Figure 10 is the tracking error curve of the memristor’s resistance to Equation (20).

It can be seen from Figure 7 and Figure 9 that at the 60th iteration, the resistance of the memristor has completely tracked the desired resistance in the time interval [0, 4] s, rather than the asymptotic tracking with time. At the same time, it can also be seen from Figure 8 and Figure 10 that the tracking error gradually converges to 0 with the increase of the number of iterations. Therefore, the control algorithm proposed in this paper can make the resistance change according to the desired trajectory. The reason is that the input voltage can approach the desired voltage step by step through the control system in Figure 2 and the iterative learning control algorithm (10). As a result, the resistance of the memristor tends to the desired trajectory, step by step.

**Figure 11:**
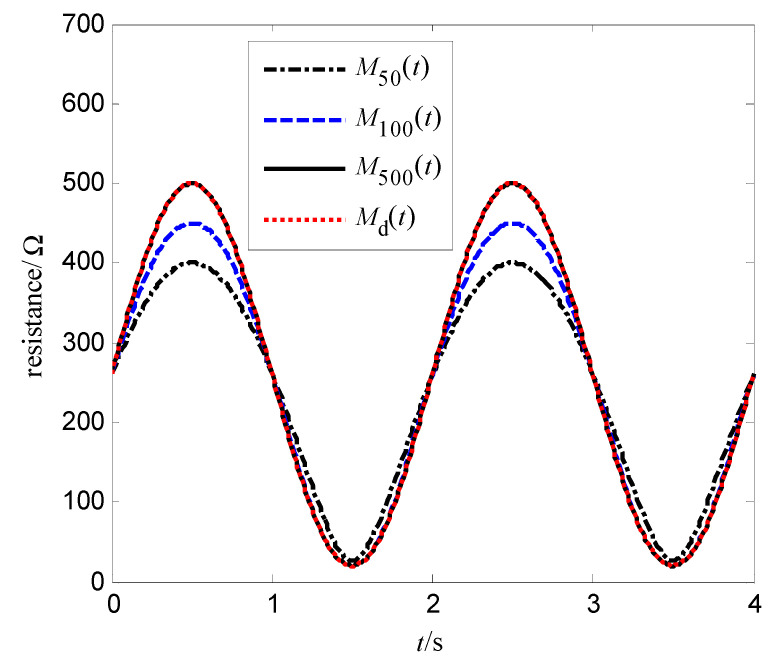
Tracking of the memristor (2) and (4) to desired resistance.

**Figure 12:**
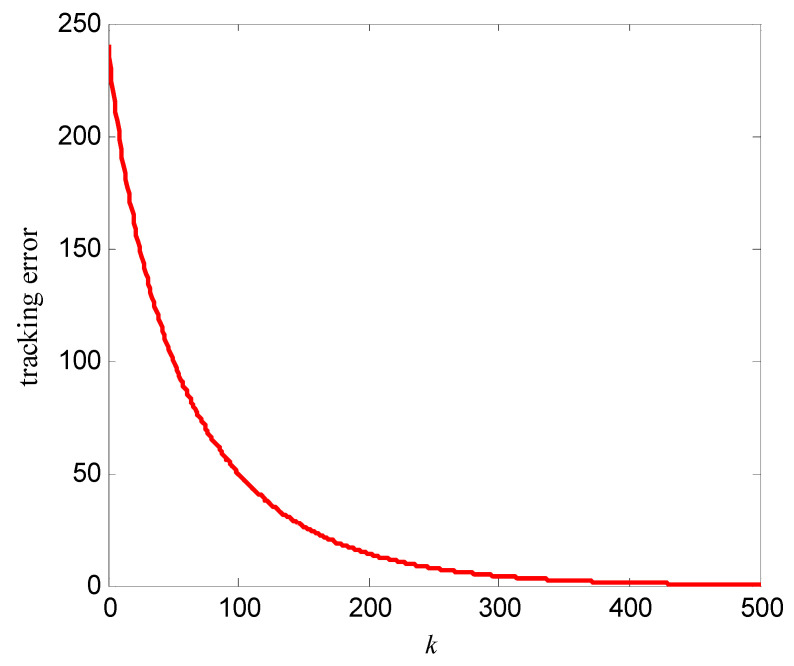
Tracking error of the memristor (2) and (4) to desired resistance.

In order to further verify the effectiveness of this algorithm, the memristor model represented by Equations (2) and (4) is simulated here. Its simulation parameters are set as follows: $R_{\mathrm{on}}=100\;\Omega$, $R_{\mathrm{off}}=600\;\Omega$, $\mu_{v}=10^{-14}\;m^{2}s^{-1}V^{-1}$, D=10 nm, the iterative learning gain L=−0.00001, the control voltage at the first iteration $v_{0}(t)=0$, the desired resistance of memristor $M_{d}(t)=260+240\sin(\pi t)$, the initial resistance $M(0)=M_{d}(0)=260\;\Omega$, and the simulation time T=4 s. The simulation results are shown in Figure 11 and Figure 12. Among them, Figure 11 is the tracking curve at the 50th, 100th, and 500th iteration learning, and Figure 12 is the convergence curve of the tracking error with the iterations. As can be seen from Figure 11 and Figure 12, the algorithm (10) in this paper can also realize the resistance tracking control for the memristor model represented by Equations (2) and (4).

Hardware-adjustable resistors in engineering mainly include rheostats, resistance boxes, and programmable resistors. The variation of the resistance value of these traditional hardware-adjustable resistors is discrete, and the accuracy of the resistance value depends on the set block, the number of resistors, or the number of network nodes. However, the variable resistance designed by memristor and iterative learning control method in this paper can be adjusted continuously and can track the desired resistance with arbitrary precision. Therefore, compared with the traditional hardware-adjustable resistor, the adjustable resistor designed in this paper can improve the continuity, flexibility, and adjusting accuracy of the variable resistor.

## 6. Conclusions

Aiming at how to make the resistance of the memristor change according to the desired trajectory, this paper proposes an iterative learning control method for the resistance of the memristor by analyzing the general mathematical model of the voltage-controlled memristor. The method is to use successive iterative learning to make the control voltage of the memristor gradually approach the desired voltage with the increase of the iterations, achieving the purpose of changing the resistance of the memristor according to the desired resistance. Furthermore, the convergence of the proposed algorithm is mathematically strictly proved, and the convergence conditions are deduced. The research results show that the proposed algorithm can make the resistance of the memristor completely track the desired resistance in a finite time interval with the increase of iterations. The controller based on this method not only has a simple structure that is convenient for engineering implementation, but it does not rely on the precise mathematical model of the memristor. At the same time, compared with the traditional digital potentiometer, the resistance control system designed in this paper can improve the continuity, flexibility, and accuracy of variable resistor, which will lay a foundation for the application research on memristors in analog circuits, digital circuits, and other fields.

## Data Availability

Not applicable.
